# Latent profile analysis of university students’ self-management and self-monitoring in the links among motivation, engagement, and wellbeing

**DOI:** 10.3389/fpsyg.2022.1023920

**Published:** 2022-11-08

**Authors:** Rui Zhao, Tianye Ling

**Affiliations:** ^1^School of Economics and Management, Zhejiang Ocean University, Zhoushan, China; ^2^Department of Education, College of Education, Hanyang University, Seoul, South Korea

**Keywords:** self-directed learning, higher education, self-management, self-monitoring, latent profile analysis

## Abstract

This study drew on Garrison’s self-directed learning model for university students in a self-determination theory framework. We adopted a person-centered approach to explore the different combinations of self-management and self-monitoring. Using a sample of Chinese university students (*N* = 142), we obtained the following data *via* a self-report survey: autonomous motivation, controlled motivation, self-management, self-monitoring, academic engagement, and wellbeing. Latent profile analysis (LPA) distinguished three self-management and self-monitoring profiles, which are “very low/low,” “high/high,” and “low/very low.” Profiles with a high level of self-management and self-monitoring were positively connected with adaptive outcomes and linked to autonomous motivation. Implications are outlined for theory and practice.

## Introduction

As a traditional learning theory, self-directed learning (SDL) plays a vital role in formal, informal, and unformal learning (e.g., Abd-El-Fattah, 2010; Kim et al., 2019; Zhu et al., 2020). Merriam et al. (2007) emphasized that a critical assumption in SDL is that “people take the primary initiative for planning, carrying out, and evaluating their own learning experiences” (p. 110). Transitioning from high school to university is a critical challenge for students, which means profound changes in life and learning (Quan et al., 2014). For most university students, this was their first experience living away from their families and learning independently. Moreover, high school classes, where preparation for the college entrance exam takes a large part, tend to focus heavily on delivering and acquiring specific knowledge in line with the competition for higher education (Sablina et al., 2018). On the contrary, university classes emphasize acquiring knowledge through the active participation of students rather than requiring students to acquire specific knowledge passively (Garn and Morin, 2021). SDL is therefore highly significant for university students.

Prior SDL research has mainly focused on the SDL process’s external factors (e.g., external management) rather than internal factors, such as cognitive processing. To cover this gap, Garrison (1997) proposed an SDL comprehensive model that includes motivation (entering/task), self-management (control), and self-monitoring (responsibility). Garrison’s SDL model emphasizes that learners’ motivation could enhance their self-management and self-monitoring. Meanwhile, self-management and self-monitoring reflect the complex integration of external factors and cognitive processing, which could enhance SDL. A growing body of studies has examined Garrison’s SDL model; however, these studies typically employed a variable-centered approach instead of a person-centered approach (Zhu et al., 2020). While the variable-centered approach has been proven valuable in identifying linear correlations, it is difficult to describe the complicated interaction between self-management and self-monitoring (Bergman and Andersson, 2010). Moreover, in self-determination theory, motivation is defined as a quality way, which varies from high autonomy to high control, and has different functions for learners’ learning and psychological outcomes (Ryan and Deci, 2000). In other words, the combinations of the quality and quantity dimensions of motivation jointly contribute to students’ learning and psychological state. Prior studies did not adopt a quality view of motivation that prevents teachers, researchers, and policy creators from understanding which kind of motivation matters for students’ SDL. Also, researchers mainly focused on the factors that improve students’ SDL, not the benefits of the SDL for students’ learning and psychological outcomes, such as engagement and wellbeing (Zhu et al., 2020).

**FIGURE 1:**
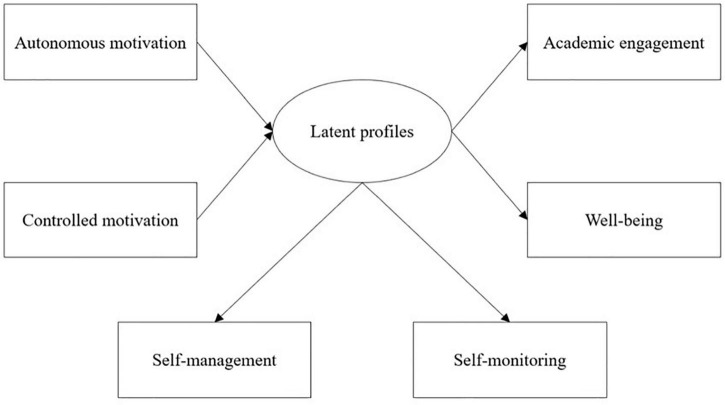
Research model.

These research gaps have prevented us from understanding the different patterns of the interaction between learners’ self-management and self-monitoring and the distinct prediction effects of motivation in the SDL context. Through a person-centered lens, we can investigate the sub-populations of the different combination patterns of self-management and self-monitoring to provide learners with suitable support. In this study, we used latent profile analysis (LPA), a model-based method (Pastor et al., 2007), to understand the different combinations of self-management and self-monitoring within individuals. In addition, we explored how different types of motivation predict self-management and self-monitoring profiles and whether differences existed in engagement and wellbeing with different self-management and self-monitoring profiles. The research model for this study is presented in Figure 1. The research questions are formulated as follows:

Q1. How can university students be classified according to their self-management and self-monitoring?

Q2. How do different types of motivation predict profile membership?

Q3. Do differences exist between the identified profiles with respect to their engagement and wellbeing?

### Self-directed learning: Garrison’s self-directed learning model

**FIGURE 2:**
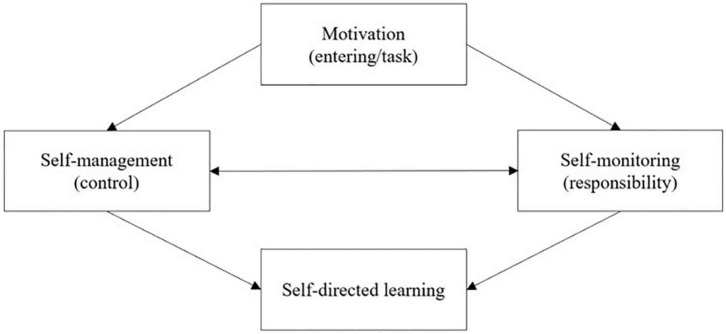
Garrison’s SDL model.

SDL is initially proposed by Tough (1971), and since then, it has been well researched in informal learning. Recently, more and more researchers have emphasized the importance of SDL in formal learning (e.g., school education and higher education; Abd-El-Fattah, 2010; El-Gilany and Abusaad, 2013; Maltais et al., 2021; Chen et al., 2022). The researchers proposed different theoretical models to account for the SDL based on their theoretical statements (Merriam et al., 2007). In 1997, Garrison put forward a multi-dimensional SDL model (see Figure 2) based on a “collaborative constructivist.”

Garrison’s SDL model includes three dimensions, which are “motivation (entering/task),” “self-management (control),” and “self-monitoring (responsibility).” Self-management is related to task contexts that are the external control of learning activities. This dimension is about setting learning goals and managing learning resources and support. According to Garrison (1997), self-management can contribute to adaptive learning outcomes in the learning process. The second dimension of Garrison’s SDL model is self-monitoring. Self-monitoring reflects the cognitive and metacognitive components of the learning process. Self-monitoring focuses on learners who take the responsibility to monitor their learning activities. The third dimension of Garrison’s SDL model is motivation. Motivation is related to the initiation and maintenance of the learning process. Garrison (1997) stated that motivation could be divided into entering and task motivation. Entering motivation is related to the tendency of learners to participate in the learning process. Task motivation is associated with continuity and focus on the learning process.

In Garrison’s SDL model, self-management and self-monitoring are distinct but theoretically reciprocal. Recent studies have tested Garrison’s SDL model’s validity in different populations and learning contexts (Abd-El-Fattah, 2010; Zhu et al., 2020). For example, Abd-El-Fattah (2010) proved that self-management positively affects self-monitoring. Zhu et al. (2020) confirmed that self-monitoring facilitates self-management. However, these studies did not concern the complex reciprocal relationship between self-management and self-monitoring. Given these findings, examining the interplay of self-management and self-monitoring might have gone unnoticed because prior studies hypothesized the unidirectional relationship between these two distinct but corrected variables. To cover this research gap, we employed a person-centered approach to examine the interplay of self-management and self-monitoring within individuals. Moreover, these studies proved a high correlation between self-management and self-monitoring (Abd-El-Fattah, 2010; Zhu et al., 2020). Garrison (1997) also emphasized that high levels of self-management may prevent learners from acquiring high levels of self-monitoring and adaptive learning outcomes. Therefore, in Garrison’s (1997) point, it may not exist the high self-management and high self-monitoring profiles. Meanwhile, people with high self-management may have less desired outcomes than others.

### Quality of motivation: A self-determination theory perspective

Motivation explains the reasons humans start and insisting behavior. Prior studies have been regarding motivation as a quantity wary. The quantity of motivation determines whether humans start or stop the behavior. However, based on empirical evidence, SDT explains motivation in a quality way, a continuum from high autonomy to high control (Ryan and Deci, 2000). Autonomous motivation means that people participate in activities because they think it is interesting (intrinsic motivation), view it as congruent with their value (integrated motivation), or believe it is essential (identified motivation). Controlled motivation means that people engage in activities because they do not want to feel guilt or shame (introjected motivation) or avoid punishment (external motivation). Intrinsic motivation is the healthiest motivation, representing high integrated levels. Recently, some researchers argued that Ryan and Deci’s (2000) motivation scale could not reflect a continuum from control to autonomy. Moreover, integrated motivation is difficult to be measured (Sheldon et al., 2017). Therefore, Sheldon et al. (2017) suggested a new motivation scale that includes intrinsic motivation, identified motivation, positive introjected motivation, negative introjected motivation, and external motivation. In this new motivation scale, autonomous motivation consists of intrinsic and identified motivation; controlled motivation consists of positive introjected, negative introjected, and external motivation. Researchers have proven that Sheldon et al. (2017)’ motivation scale is better than Ryan and Deci’s (2000) original motivation scale.

In SDT, autonomous motivation enhances adaptive outcomes (Ryan and Deci, 2000), while controlled motivation leads to maladaptive outcomes. Although Garrison (1997) emphasizes the importance of intrinsic motivation in “meaningful and worthwhile learning,” Garrison’s SDL model did not consider motivation in a quality way, leading to it not distinguishing the distinct effects of different kinds of motivation. In sum, we hypothesize that autonomous motivation will relate to more adaptive self-management and self-monitoring profiles. Also, the controlled motivation will relate to more maladaptive profiles. Redefine Garrison’s SDL model in an SDT framework attribute to distinguish the functions of motivation in the SDL process.

### The present study

This study aimed to extend Garrison’s SDL model in a combination of quality and quantity views of motivation. Meanwhile, self-management and self-monitoring are reciprocally related, but in Garrison’s (1997) opinion, self-management may facilitate self-monitoring and desired outcomes. Considering the complex relationship between self-management and self-monitoring, it is hard to describe this relationship in a variable-centered approach (e.g., linear regression analysis). To cover this research gap, we employ a person-centered approach to describe the interplay of self-management and self-monitoring within individuals. Moreover, the initial aim of Garrison’s SDL model is to describe the SDL process in informal and unformal learning contexts. It did not explain whether university students can benefit from SDL. Zhu et al. (2020) suggested that future research could examine the effects of SDL on engagement. Meanwhile, wellbeing reflects a positive psychological state. Thus, we choose engagement and wellbeing as the outcomes of self-management and self-monitoring profiles.

To achieve the purpose of this study, first, we decided on the optimal number of self-management and self-monitoring profiles, second, we examined the distinct effects of autonomous motivation and controlled motivation on self-management and self-monitoring profiles, and finally, we explored the differences in engagement and wellbeing across different self-management and self-monitoring profiles.

## Materials and methods

### Participants

To achieve the purpose of this study, we conducted an online survey *via* the Wenjuanxing platform,^1^ a web-based questionnaire platform in China. Specifically, the questionnaire’s OR code was sent to potential participants who were university students, and all items were marked as necessary. To ensure these participants do not randomly select the answers, we set a wrong item (“I do not have a telephone”). A total of 150 participants scanned the OR code and completed the online survey. Excluding eight university students, the remaining 142 university students (66 males and 76 females) from different universities in China responded to all items.

### Measures

The measures were translated from English to Chinese by the researcher, a native Chinese speaker and fluent in English. A back-translation from Chinese to English was conducted by a native Chinese speaker fluent in Chinese. Throughout the questionnaire, we used the same 1–5 Likert response scale (1 = strongly disagree; 5 = strongly agree).

#### Motivation

To assess different types of motivation, we used a 25-item questionnaire developed by Sheldon et al. (2017). Intrinsic motivation (e.g., “I study because I enjoy it”; Cronbach’s coefficient alpha = 0.770), identified motivation (e.g., “I study because I strongly value it”; Cronbach’s coefficient alpha = 0.788), positive introjected motivation (e.g., “I study because I want to feel proud of myself”; Cronbach’s coefficient alpha = 0.725), negative introjected motivation (e.g., “I study because I would feel guilty if I did not do it”; Cronbach’s coefficient alpha = 0.725), and external motivation (e.g., “I study because important people (i.e., parents, professors) will like me better if I do it”; Cronbach’s coefficient alpha = 0.675) were assessed by five items, respectively.

#### Self-management and self-monitoring

We assessed self-management and self-monitoring by adapting the SDL readiness scale (Fisher and King, 2010). Self-management (e.g., “I set strict time frames”; Cronbach’s coefficient alpha = 0.825) and self-monitoring (e.g., “I prefer to set my own learning goals”; Cronbach’s coefficient alpha = 0.746) were assessed by 10 items, respectively.

#### Engagement

We assessed four components (behavior, emotion, cognition, and agency) of engagement. To assess the behavioral engagement and emotional engagement, we used the five-item behavioral engagement scale (e.g., “I try hard to do well in school”; Cronbach’s coefficient alpha = 0.847) and five-item emotional engagement scale (e.g., “When I’m in class, I feel good”; Cronbach’s coefficient alpha = 0.847), which were developed by Skinner et al. (2009). To assess cognitive engagement, we used the four-item cognitive engagement scale (e.g., “When studying for this class, I try to generate my own examples of the concepts to help me understand them better”; Cronbach’s coefficient alpha = 0.840) as suggested by Senko and Miles (2008). To assess agentic engagement, we used the five-item agentic engagement scale (e.g., “During class, I ask questions to help me learn”; Cronbach’s coefficient alpha = 0.646), which was developed by Reeve (2013).

#### Wellbeing

To assess wellbeing, we used the subjective vitality scale (Ryan and Frederick, 1997). The subjective vitality scale includes seven items (e.g., “I feel alive and vital”; Cronbach’s coefficient alpha = 0.841).

### Data analysis

We conducted LPA by Mplus 8.3 and decided the optimal numbers of profiles by Akaike information criteria (AIC), Bayesian information criteria (BIC), sample-size-adjusted BIC (aBIC), entropy, Lo–Mendell–Rubin adjusted LRT test (aLMR), and Vuong–Lo–Mendell–Rubin likelihood ratio test (VLMR). AIC, BIC, and aBIC are based on the model log-likelihood, and the lowest scores represent the preferred model. Entropy represents the precision of the cases classified into the profiles. aLMR and VLMR compare the k-profile model with the k-1 profile model. The significance of aLMR and VLMR representing the k-profile model is better than the k-1 profile model.

After identifying the optimal numbers of profiles, we standardized indicators by z-scores. We used the three-step method to examine the effects of motivation on self-management and self-monitoring profiles. We employed the BCH method to examine the effects of self-management and self-monitoring profiles on engagement and wellbeing. Although, there is no clear standardization of the magnitude of indicators. We considered values of over ± 1 SD as very high/low, values of ± 0.5 to 1 SD as high/low, and values up to ± 0.5 SD as slightly above/below average.

## Results

### Preliminary analysis

Descriptive statistics and bivariate correlations among all measured variables are given in Table 1. Self-management and self-monitoring were positively related to intrinsic motivation, identified motivation, positive introjected regulation, negative introjected regulation, behavioral engagement, emotional engagement, cognitive engagement, agentic engagement, and wellbeing. Meanwhile, self-management and self-monitoring were negatively related to external motivation.

**TABLE 1:** Descriptive statistics and correlations among variables.

	IM	IDM	PIM	NIM	EM	SM	SMO	BE	EE	CE	AE	WB
IM	1											
IDM	0.553***	1										
PIM	0.381***	0.570***	1									
NIM	0.410***	0.604***	0.725***	1								
EM	−0.456***	−0.181*	0.052	0.006	1							
SM	0.778***	0.658***	0.428***	0.477***	−0.464***	1						
SMO	0.726***	0.667***	0.505***	0.552***	−0.280**	0.790***	1					
BE	0.739***	0.630***	0.340***	0.407***	−0.502***	0.798***	0.755***	1				
EE	0.770***	0.582***	0.331***	0.364***	−0.556***	0.820***	0.681***	0.806***	1			
CE	0.352***	0.555***	0.606***	0.642***	0.078	0.462***	0.443***	0.325***	0.374***	1		
AE	0.794***	0.547***	0.371***	0.361***	−0.329***	0.742***	0.686***	0.739***	0.744***	0.3660***	1	
WB	0.783***	0.583***	0.397***	0.445***	−0.503***	0.865***	0.768***	0.829***	0.809***	0.3260***	0.747***	1
M	3.831	4.063	4.097	4.136	3.114	3.825	3.739	3.739	3.707	4.049	3.847	3.849
SD	0.845	0.611	0.698	0.674	0.744	0.701	0.581	0.928	0.887	0.665	0.797	0.822

IM, Intrinsic motivation; IDM, Identified motivation; PIM, Positive introjected motivation; NIM, Negative introjected motivation; EM, External motivation; SM, Self-management; SMO, Self-monitoring; BE, Behavioral engagement; EE, Emotional engagement; CE, Cognitive engagement; AE, Agentic engagement; WB, Wellbeing.

*p < 0.05; **p < 0.01; *** *p* < 0.001.

### Self-management and self-monitoring profiles

We decided three profiles as the optimal numbers of profiles based on a range of statistical criteria presented in Table 2. Except the aBIC was slightly higher than the four-profile model, the three-profile model’s AIC, BIC, and aBIC were lower than other models. The *p*-values of aLMR and VLMR for the three-profile model were significant, while the four-profile model was not.

**TABLE 2:** Fit indices, entropy, and model comparisons for estimated latent profile analysis model (*N* = 142).

	AIC	BIC	aBIC	Entropy	aLMR	*p* aLMR	VLMR	*p* VLMR	*N* for each profiles
1	556.873	568.697	556.041						
2	310.475	331.166	309.017	0.990	236.492	0.0000	–274.437	0.0000	53, 89
3	292.475	322.033	290.392	0.983	22.487	0.0002	–148.237	0.0001	48, 89, 5
4	291.584	330.010	288.877	0.897	6.456	0.1895	–136.237	0.1708	28, 5, 88, 21

AIC, Akaike information criteria; BIC, Bayesian information criteria; aBIC, Sample-size-adjusted BIC; aLMR, Lo–Mendell–Rubin adjusted LRT test; p aLMR, *p*-value for Lo–Mendell–Rubin adjusted LRT test; VLMR, Vuong–Lo–Mendell–Rubin likelihood ratio test; p VLMR, *p*-value for Vuong–Lo–Mendell–Rubin likelihood ratio test.

**FIGURE 3:**
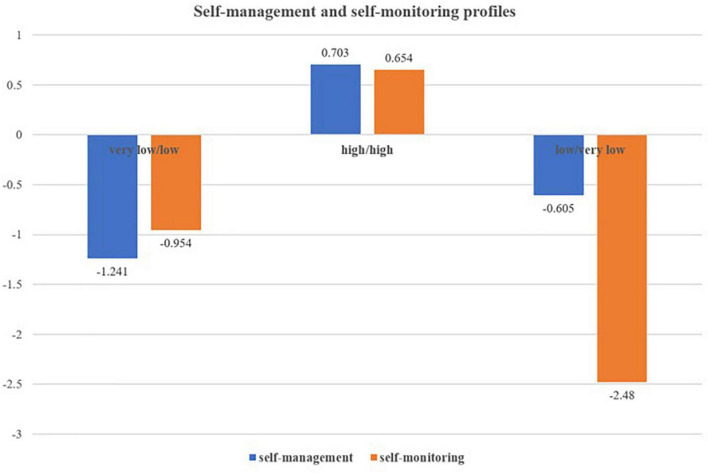
Self-management and self-monitoring profiles (*z*-score).

Figure 3 shows a graphical representation of the three profiles. Students in profile 1 showed very low self-management and low self-monitoring, so we called this “very low/low profile.” Students in profile 2 showed high self-management and high self-monitoring, so this profile was called “high/high profile.” Students in profile 3 showed low self-management and very low self-monitoring, so this profile was called “low/very low profile.”

### Antecedents of self-management and self-monitoring profiles

The effects of motivation on self-management and self-monitoring profiles are presented in Table 3. Comparing “low/very low profile” and “very low/low profile,” there were no significant predictive effects of motivation. Comparing “low/very low profile” and “high/high profile,” the identified motivation (B = 3.830*, S.E. = 1.917, OR = 46.019) and intrinsic motivation (B = 2.959^***^, S.E. = 1.035, OR = 19.275) positively affect the “high/high profile.” Meanwhile, comparing “very low/low profile” and “high/high profile,” the identified motivation (B = 2.896^**^, S.E. = 1.077, OR = 18.095) and intrinsic motivation (B = 1.945^**^, S.E. = 0.609, OR = 6.992) also positively affect the “high/high profile”.

**TABLE 3:** Results of the effects of motivation on self-management and self-monitoring profiles.

	Low/very low vs. very low/low			Low/very low vs. high/high			Very low/low vs. high/high		
			
	B	S.E.	OR	B	S.E.	OR	B	S.E.	OR
IM	1.014	1.038	2.757	2.959***	1.035	19.275	1.945**	0.609	6.992
IDM	0.933	1.401	2.543	3.830*	1.917	46.019	2.896**	1.077	18.095
PIM	0.916	0.610	2.499	0.822	2.148	2.274	–0.094	2.228	0.910
NIM	0.583	0.671	1.791	1.747	1.458	5.735	1.164	1.317	3.202
EM	1.753	1.614	5.771	0.262	2.267	1.300	–1.491	1.372	0.225

IM, Intrinsic motivation; IDM, Identified motivation; PIM, Positive introjected motivation; NIM, Negative introjected motivation; EM, External motivation.

*p < 0.05; **p < 0.01; *** *p* < 0.001.

### Consequences of self-management and self-monitoring profiles

**FIGURE 4:**
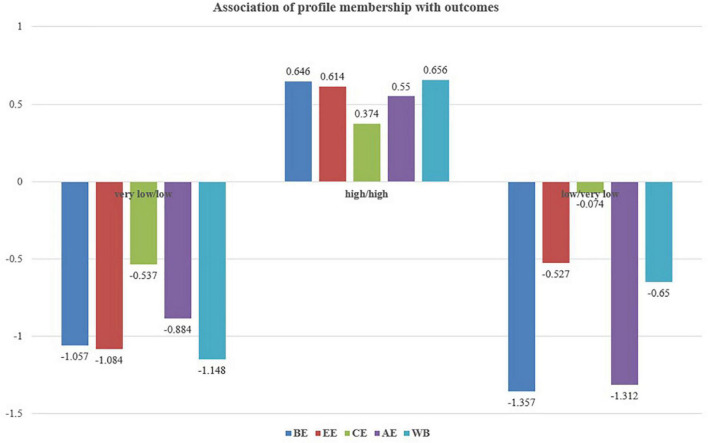
Association of profile membership with outcomes.

Turning to the outcome, the differences in the mean level of engagement and wellbeing were tested. The mean levels of each outcome across the three-profile model and the statistical significance are presented in Table 4 and Figure 4. Behavioral engagement, emotional engagement, cognitive engagement, agentic engagement, and wellbeing were highest in the “high/high profile.” However, the differences in these outcomes between the “very low/low profile” and “low/very low profile” were not statistically significant.

**TABLE 4:** Results of the effects of self-management and self-monitoring profiles on engagement and wellbeing (z-score).

	Very low/low	High/high	Low/very low
BE	–1.057^b^ (0.745)	0.646^a^ (0.368)	–1.357^b^ (0.870)
EE	–1.084^b^ (0.759)	0.614^a^ (0.480)	–0.527^b^ (0.755)
CE	–0.537^b^ (1.228)	0.374^a^ (0.519)	–0.074^b^ (1.490)
AE	–0.884^b^ (0.944)	0.550^a^ (0.524)	–1.312^b^ (0.687)
WB	–1.148^b^ (0.686)	0.656^a^ (0.392)	–0.650^b^ (0.643)

BE, Behavioral engagement; EE, Emotional engagement; CE, Cognitive engagement; AE, Agentic engagement; WB, Wellbeing.

## Discussion

This study aimed to extend the understanding of the relationships between self-management and self-monitoring, which are essential elements in SDL. Moreover, we examined the antecedents and consequences of self-management and self-monitoring profiles.

We decided three profiles as the optimal numbers of groups based on a range of statistical criteria. Due to the high correlations between self-management and self-monitoring, we only found the “very low/low profile,” “high/high profile,” and “low/very low profile.” We did not find a profile with high self-management and low self-monitoring, or reverse. It was opposite to the opinion of Garrison (1997) that high self-management may not facilitate self-monitoring but corresponds with Abd-El-Fattah’s (2010) results. After identifying optimal profiles, we tested the antecedents and consequences of the self-management and self-monitoring profiles.

We found that students with intrinsic and identified motivation were more likely to be in the “high/high profile.” However, other kinds of motivations were not mattered. That is, these kinds of motivations did not make sense for the prediction of profiles. This result proved the view of SDT that not every kind of motivation matters. Autonomous motivation makes students become more self-management and self-monitoring. However, despite controlled motivation not making students less self-management and self-monitoring, it did not make students become high self-management or high self-monitoring. It emphasized the importance of autonomous motivation. Moreover, we found that identified motivation is more likely to make students have high levels of self-management and self-monitoring than identified motivation. In SDT, intrinsic motivation is the healthiest type of motivation (Ryan and Deci, 2000). However, we examined self-management and self-monitoring in a formal learning context. University students were given established learning content and learning plans. It may need students’ identified motivation more than intrinsic motivation to integrate external conditions.

Finally, we examined the differences in engagement and wellbeing among different self-management and self-monitoring profiles. We found that students with “high/high profile” have higher engagement and wellbeing than others. It proved that self-management and self-monitoring are important for students’ engagement and wellbeing. Moreover, this result was opposite to the opinion of Garrison (1997) that high self-management may not facilitate adaptive outcomes.

### Implications for theory and practice

Our study offers theoretical and practical implications for the study of SDL. This study integrated Garrison’s SDL model and self-determination theory through a person-centered lens. Our findings revealed that while the relations between self-management and self-monitoring are regarded as unidirectional in the prior studies (Abd-El-Fattah, 2010; Zhu et al., 2020), LPA provided evidence for the bidirectional relations between self-management and self-monitoring. Specifically, the existence of the very low/low and high/high profiles of self-management and self-monitoring highlights the reciprocal relationship between self-management and self-monitoring. The enhancement of self-management may contribute to the high levels of self-monitoring. Meanwhile, self-monitoring also promotes the development of self-management.

The application of these findings has the potential to enhance educational practices. Although university students are given established learning content and learning plans in the formal learning contexts, they can have high levels of self-management and self-monitoring, which matter for their learning and psychological outcomes. It emphasizes the demand to enhance university students’ SDL in formal learning contexts. Our study also showed that the combination of the quality and quantity dimensions of motivation matter for SDL. In other words, the quantity of controlled motivation does not matter for students’ SDL. As teachers, they can enhance students’ intrinsic and identified motivation to help students become more engaged and happier in the learning process.

### Limitations and future directions

As for the limitations, first, we only used 142 university students to identify the profiles of self-management and self-monitoring, which may prevent us from finding more profiles and cannot ensure the validity of the results. Future research needs to put forward the study in large size of participants. Second, the relationships between motivation, self-management and self-monitoring profiles, engagement, and wellbeing were explored through cross-sectional research design. Therefore, no causal relationships could be inferred. Future research can take a longitudinal design to cover this gap. Finally, due to the high positive correlations between self-management and self-monitoring, we did not find high/low profiles. Future research can add some other elements of SDL to explore different kinds of profiles.

## Data availability statement

The original contributions presented in this study are included in the article/supplementary material, further inquiries can be directed to the corresponding author.

## Author contributions

Both authors listed have made a substantial, direct, and intellectual contribution to the work, and approved it for publication.

## Footnotes

1
https://www.wjx.cn/
